# Use of brain MRI and gene expression atlases to reconstruct the pathophysiology of autoimmune neurological disorders: The proof-of-concept of NMOSD

**DOI:** 10.1177/13524585241307154

**Published:** 2024-12-31

**Authors:** Laura Cacciaguerra, Loredana Storelli, Elisabetta Pagani, Paolo Preziosa, Sharlota Mesaros, Vittorio Martinelli, Lucia Moiola, Marta Radaelli, Jovana Ivanovic, Olivera Tamas, Jelena Drulovic, Massimo Filippi, Maria A Rocca

**Affiliations:** Neuroimaging Research Unit, Division of Neuroscience, IRCCS San Raffaele Scientific Institute, Milan, Italy; Vita-Salute San Raffaele University, Milan, Italy; Neuroimaging Research Unit, Division of Neuroscience, IRCCS San Raffaele Scientific Institute, Milan, Italy; Neuroimaging Research Unit, Division of Neuroscience, IRCCS San Raffaele Scientific Institute, Milan, Italy; Neuroimaging Research Unit, Division of Neuroscience, IRCCS San Raffaele Scientific Institute, Milan, Italy; Vita-Salute San Raffaele University, Milan, Italy; Neurology Unit, IRCCS San Raffaele Scientific Institute, Milan, Italy; Neurology Clinic, University Clinical Centre of Serbia, Faculty of Medicine, University of Belgrade, Belgrade, Serbia; Neurology Unit, IRCCS San Raffaele Scientific Institute, Milan, Italy; Neurology Unit, IRCCS San Raffaele Scientific Institute, Milan, Italy; Neurology Unit, IRCCS San Raffaele Scientific Institute, Milan, Italy; Neurology Clinic, University Clinical Centre of Serbia, Belgrade, Serbia; Neurology Clinic, University Clinical Centre of Serbia, Belgrade, Serbia; Neurology Clinic, University Clinical Centre of Serbia, Faculty of Medicine, University of Belgrade, Belgrade, Serbia; Neuroimaging Research Unit, Division of Neuroscience, IRCCS San Raffaele Scientific Institute, Milan, Italy; Vita-Salute San Raffaele University, Milan, Italy; Neurology Unit, IRCCS San Raffaele Scientific Institute, Milan, Italy; Neurorehabilitation Unit, IRCCS San Raffaele Scientific Institute, Milan, Italy; Neurophysiology Service, IRCCS San Raffaele Scientific Institute, Milan, Italy; Neuroimaging Research Unit, Division of Neuroscience, IRCCS San Raffaele Scientific Institute, Milan, Italy; Vita-Salute San Raffaele University, Milan, Italy; Neurology Unit, IRCCS San Raffaele Scientific Institute, Milan, Italy

**Keywords:** Neuromyelitis optica spectrum disorders, magnetic resonance imaging, gene expression

## Abstract

**Background:**

The understanding of disease pathophysiology is pivotal for tailored treatments. The spatial distribution of brain damage relies on the regional antigen expression and the local balance of susceptibility and protective elements.

**Objective:**

As proof-of-concept, we investigated the spatial association between brain damage and gene expression in aquaporin-4-IgG-positive neuromyelitis optica spectrum disorder (AQP4 + NMOSD).

**Methods:**

In this multicenter cross-sectional study, 90 AQP4 + NMOSD patients and 94 age-matched healthy controls underwent a brain magnetic resonance imaging (MRI). We used T2-hyperintense lesion probability maps and white/gray matter atrophy as proxies of inflammation and neurodegeneration. The association with the expression of 266 candidate genes was obtained with the Multimodal Environment for Neuroimaging and Genomic Analysis platform. A functional-enrichment analysis investigated overrepresented biological processes.

**Results:**

In AQP4 + NMOSD, T2-hyperintense lesions were mainly periventricular; atrophy mostly involved the visual pathway. The expression of AQP4 and complement (C4a and C5) was associated with both inflammation and neurodegeneration. Complement activation and regulation/uptake of the insulin-like growth factor were the most relevant enriched pathways. Nonspecific pathways related to DNA synthesis and repair were associated with brain atrophy.

**Conclusions:**

Quantitative MRI and gene expression atlas identified the key elements of AQP4 + NMOSD pathophysiology. This analysis could help in understanding the pathophysiology of antibody-mediated autoimmune disorders.

## Introduction

As demonstrated in aquaporin-4 (AQP4)-IgG positive neuromyelitis optica spectrum disorder (AQP4 + NMOSD), knowledge of the pathophysiology of autoimmune neurological conditions is the most important step for the development of tailored treatments.^1,2^

Summarizing, a pro-inflammatory environment and the peripheral loss of self-tolerance lead to the production of autoreactive IgGs by plasmablasts. These antibodies enter the central nervous system (CNS) and bind to the AQP4 water channel expressed on the astrocytes end-feet, where they elicit the classical pathway of the complement cascade with primary astrocytes’ damage. Collateral local recruitment of granulocytes determines secondary injury to the bystander tissues.^3^

This model was proven by the extraordinary success of the phase III randomized clinical trials of drugs targeting AQP4 + NMOSD pathophysiological pillars: inflammation (i.e. interleukin-6 (IL-6) receptor inhibitors),^4,5^ antibody production (i.e. anti-CD19),^6^ and complement (i.e. complement inhibitors).^7,8^

Antibody-associated autoimmune conditions target antigens with a different expression across CNS regions. Spatial distribution of damage is likely dependent on both regional antigen expression and local balance between protective and susceptibility factors. Preclinical studies demonstrated that AQP4 expression in tissues defines the susceptibility to AQP4 + NMOSD-related damage. In fact, the expression of the AQP4 messenger RNA and proteins are significantly higher in the optic nerve and spinal cord,^9^ which are the main targets of the disease. Similarly, magnetic resonance imaging (MRI) studies showed that focal brain lesions typically locate at areas with high AQP4 expression, such as the periependymal lying and the area postrema.^10^ Atrophy has been reported as well,^11^ but less is known about the drivers of neurodegeneration in AQP4 + NMOSD.

Transcriptomic atlases enclosing quantitative gene expression at different brain locations such as the Allen Human Brain Atlas (AHBA),^12^ are currently available. They can be linked to the Montreal National Institute space (MNI), the standard reference for MRI, through novel computational tools such as the Multimodal Environment for Neuroimaging and Genomic Analysis (MENGA) platform.^13^

Nowadays, new technologies are speeding up antibody discovery, but the understanding of the underlying pathophysiology and consequent identification of therapeutic targets is challenging.

In this study, we tried to establish whether the joint application of quantitative MRI and human brain gene expression atlases can underpin key elements of disease pathogenesis through the analysis of damage localization. Given the knowledge of its pathophysiology, we used AQP4 + NMOSD as a proof-of-concept disorder. In this analysis, CNS damage caused by inflammation and neurodegeneration were represented by T2-hyperintense lesions and regional atrophy, respectively.

## Materials and methods

### Participants

We included brain MRI scans from 80 consecutive AQP4 + NMOSD patients enrolled in our research protocol. Patients were diagnosed according to the 2015 diagnostic criteria^14^ (AQP4-IgG tested using a cell-based assay)^14^ and were clinically stable for at least 1 month before the MRI acquisition. A total of 94 age- and scanner-matched healthy controls (HC) were also included.

Two European centers were involved: Milan (92 subjects: 40 AQP4 + NMOSD and 52 HC) and Belgrade (82 subjects: 40 AQP4 + NMOSD and 42 HC). HC were recruited among research or hospital personnel, students, friends, and acquaintances. By definition, they did not have any major health condition, including neurological disorders. Additional exclusion criteria for all participants, as per our protocol, were alcohol or drug abuse, history of head trauma, psychiatric comorbidities, and contraindications to MRI. On the day of the MRI acquisition, patients underwent a neurological examination, including the assessment of the Expanded Disability Status Scale (EDSS)^15^ and history collection.

### MRI acquisition protocol

Using two 3.0T scanners (Milan: Ingenia CX and Intera Philips Medical Systems) and a 1.5T scanner (Belgrade: Achieva Philips Medical System), the following brain sequences were collected from all subjects in a single session:

1. T2-weighted images: axial dual-echo turbo spin-echo (TSE; Intera scanner in Milan and Belgrade) or sagittal 3D fluid attenuated inversion recovery (FLAIR; Ingenia CX scanner in Milan);
2. 3D T1-weighted images: axial 3D T1-weighted fast gradient-echo (Intera scanner in Milan), axial 3D T1-weighted turbo field echo (Belgrade), and sagittal 3D T1-weighted magnetization-prepared rapid gradient-echo (Ingenia scanner in Milan).

Details of the MRI protocols are available in Table 1.

**Table 1.** Detailed description of the standardized MRI protocol at each participating center and scanner.

	Scanner	Field strength (T)	Sequence	TR/TE (ms)	TI (ms)	Matrix	Thickness (mm)
T2-w	Milan, Intera	3.0	Axial dual-echo TSE	2599/16–80	–	256 × 256	3
	Milan, Ingenia	3.0	Sagittal 3D FLAIR	4800/270	1650	256 × 256	1
	Belgrade	1.5	Axial dual-echo TSE	3141/20–100	–	256 × 256	3
T1-w	Milan, Intera	3.0	Axial 3D FFE	25/4.6	–	256 × 256	0.8
	Milan, Ingenia	3.0	Sagittal 3D MPRAGE	7/3.2	1000	256 × 256	1
	Belgrade	1.5	Axial 3D TFE	7.2/3.2	1000	256 × 256	1

3D: three-dimensional; FFE: fast field echo; FLAIR: fluid attenuated inversion recovery; MPRAGE: magnetization prepared rapid gradient echo; *T*: tesla; T1-w: T1-weighted sequences; T2-w: T2-weighted sequences; TE: echo time; TFE: turbo field echo; TI: inversion time; TR: relaxation time; TSE: turbo spin-echo.

### MRI analysis

#### Conventional MRI analysis

Focal T2-hyperintense white matter lesions were segmented on T2-weighted images using a fully automated approach based on two 3D patch-wise convolutional neural networks^16^ (Milan, Ingenia scanner) or a local thresholding segmentation technique (Jim 8.0 Xinapse System Ltd.; Milan, Intera scanner, and Belgrade) to obtain lesion masks and volumes. Head-size normalized volumes of the brain, gray matter, and white matter were measured after T1-hypointense lesion refilling using the FSL SIENAx software.^17^

#### T2-hyperintense lesion probability map in AQP4 + NMOSD patients

By exploiting the transformation matrix of the voxel-based morphometry (VBM) (refer to the “VBM” section for further details), we coregistered the single-subject T2-lesion masks to the standard space, obtaining a lesion probability map (i.e. 0%–100% probability of a voxel of being involved by a lesion). Since this specific analysis only included the subgroup of AQP4 + NMOSD patients with brain lesions, and the lesion volume in AQP4 + NMOSD is usually moderate, the lesion mask of the right hemisphere was mirrored on the left hemisphere to strengthen statistical power (i.e. if corresponding voxels on the two hemispheres were involved by a lesion, the probability of having a lesion in that voxel was calculated as the sum of both probabilities). The choice of the left hemisphere was driven by the fact that most of the AHBA donors were sampled only in the left hemisphere.

#### VBM analysis

VBM analysis was used to identify regions of significant gray matter and white matter atrophy in AQP4 + NMOSD patients compared with HC. The opposite contrast was also performed as negative control.

3D T1-weighted images were segmented using the Segmentation tool available in SPM12 and normalized through the Diffeomorphic Anatomical Registration using Exponentiated Lie Algebra (DARTEL) registration method.^18^ Gray and white matter maps were modulated and smoothed (3D 8-mm Gaussian kernel). Between-group comparisons were run with a two-factor (i.e. AQP4 + NMOSD and HC) and three-level (i.e. scanners) ANOVA, with age, sex, and the SIENAX-derived V-scaling factor as covariates; *p* values < 0.001 were considered significant.

#### Spatial associations between image-derived maps and gene expression

Brain-wide gene expression profiles were obtained from the AHBA, which provides gene expression data for more than 20,000 genes from about 3700 spatially distinct tissue samples obtained from six neurotypical adult brains.^12^ We preselected disease-specific genes of interest using the Open Target Platform and the search term: “Neuromyelitis optica” (https://platform.opentargets.org/disease/)^19^ and obtained a list of 266 candidate genes (Table 2) included in both the AHBA database and the MENGA platform (see below).

**Table 2.** List of candidate genes analyzed in the study with the overall association score (i.e. degree of association with AQP4 + NMOSD according to the Open Target Platform).

Ranking	Symbol	Overall association score	Target name
1	IL6R	0.53257	Interleukin 6 receptor
2	IL6ST	0.52442	Interleukin 6 cytokine family signal transducer
3	TOP2A	0.47019	DNA topoisomerase II alpha
4	IMPDH1	0.43426	Inosine monophosphate dehydrogenase 1
5	IMPDH2	0.43426	Inosine monophosphate dehydrogenase 2
6	MS4A1	0.40109	Membrane spanning 4-domains A1
7	NR3C1	0.40043	Nuclear receptor subfamily 3 group C member 1
8	CD19	0.39164	CD19 molecule
9	C5	0.36215	Complement C5
10	PPAT	0.28540	Phosphoribosyl pyrophosphate amidotransferase
11	HLA-DQA1	0.13565	Major histocompatibility complex, class II, DQ alpha 1
12	AQP4	0.11965	Aquaporin 4
13	HLA-DRB1	0.09750	Major histocompatibility complex, class II, DR beta 1
14	IFNA1	0.08614	Interferon alpha 1
15	CSF3R	0.08316	Colony stimulating factor 3 receptor
16	MOG	0.08278	Myelin oligodendrocyte glycoprotein
17	POLA1	0.08223	DNA polymerase alpha 1, catalytic subunit
18	POLA2	0.08223	DNA polymerase alpha 2, accessory subunit
19	POLD1	0.08223	DNA polymerase delta 1, catalytic subunit
20	POLD2	0.08223	DNA polymerase delta 2, accessory subunit
21	POLD3	0.08223	DNA polymerase delta 3, accessory subunit
22	POLD4	0.08223	DNA polymerase delta 4, accessory subunit
23	POLE	0.08223	DNA polymerase epsilon, catalytic subunit
24	POLE2	0.08223	DNA polymerase epsilon 2, accessory subunit
25	POLE3	0.08223	DNA polymerase epsilon 3, accessory subunit
26	PRIM1	0.08223	DNA primase subunit 1
27	PRIM2	0.08223	DNA primase subunit 2
28	IL1B	0.08036	Interleukin 1 beta
29	SPP1	0.07957	Secreted phosphoprotein 1
30	CD38	0.07502	CD38 molecule
31	APOA1	0.07484	Apolipoprotein A1
32	CD59	0.07430	CD59 molecule (CD59 blood group)
33	GSR	0.07392	Glutathione-disulfide reductase
34	PSMB5	0.07392	Proteasome 20S subunit beta 5
35	TOP2B	0.07392	DNA topoisomerase II beta
36	CCR7	0.05802	C-C motif chemokine receptor 7
37	IL6	0.05575	Interleukin 6
38	TNXB	0.05066	Tenascin XB
39	C1S	0.04607	Complement c1s
40	KCNJ10	0.04540	Potassium inwardly rectifying channel subfamily J member 10
41	CD58	0.04396	CD58 molecule
42	GFAP	0.04074	Glial fibrillary acidic protein
43	FCRL3	0.04071	Fc receptor like 3
44	CXCL10	0.04054	C-X-C motif chemokine ligand 10
45	IL7	0.03909	Interleukin 7
46	F11	0.03696	Coagulation factor XI
47	F12	0.03696	Coagulation factor XII
48	HRH1	0.03696	Histamine receptor H1
49	KLKB1	0.03696	Kallikrein B1
50	MBP	0.03688	Myelin basic protein
51	RRM1	0.03326	Ribonucleotide reductase catalytic subunit M1
52	AIRE	0.03253	Autoimmune regulator
53	ICOS	0.03104	Inducible T cell costimulator
54	NMNAT2	0.03088	Nicotinamide nucleotide adenylyltransferase 2
55	CLIC1	0.03085	Chloride intracellular channel 1
56	GJB1	0.02938	Gap junction protein beta 1
57	CSF3	0.02801	Colony stimulating factor 3
58	IL7R	0.02716	Interleukin 7 receptor
59	RETN	0.02716	Resistin
60	CD40LG	0.02632	CD40 ligand
61	SDC1	0.02605	Syndecan 1
62	IL4	0.02520	Interleukin 4
63	MMP9	0.02493	Matrix metallopeptidase 9
64	SLC44A4	0.02455	Solute carrier family 44 member 4
65	DPYSL5	0.02370	Dihydropyrimidinase like 5
66	ALB	0.02294	Albumin
67	TGM2	0.02217	Transglutaminase 2
68	GJC2	0.02215	Gap junction protein gamma 2
69	CXCR3	0.02204	C-X-C motif chemokine receptor 3
70	S100B	0.02191	S100 calcium binding protein B
71	HLA-DPB1	0.02147	Major histocompatibility complex, class II, DP beta 1
72	CD55	0.02096	CD55 molecule (Cromer blood group)
73	IFIH1	0.02070	Interferon induced with helicase C domain 1
74	GAN	0.01982	Gigaxonin
75	CD8A	0.01892	CD8a molecule
76	TH	0.01891	Tyrosine hydroxylase
77	SLC1A2	0.01870	Solute carrier family 1 member 2
78	TIMP1	0.01848	TIMP metallopeptidase inhibitor 1
79	IFI30	0.01846	IFI30 lysosomal thiol reductase
80	CRP	0.01762	C-reactive protein
81	IRF5	0.01682	Interferon regulatory factor 5
82	CD4	0.01628	CD4 molecule
83	AKT1	0.01626	AKT serine/threonine kinase 1
84	ANGPT1	0.01626	Angiopoietin 1
85	QKI	0.01626	QKI, KH domain containing RNA binding
86	CD226	0.01608	CD226 molecule
87	RELA	0.01571	RELA proto-oncogene, NF-kb subunit
88	C4A	0.01524	Complement C4A (Rodgers blood group)
89	NEFL	0.01515	Neurofilament light chain
90	BECN1	0.01478	Beclin 1
91	CCL7	0.01478	C-C motif chemokine ligand 7
92	MIF	0.01404	Macrophage migration inhibitory factor
93	IL22	0.01384	Interleukin 22
94	CCL4	0.01330	C-C motif chemokine ligand 4
95	IL32	0.01277	Interleukin 32
96	NGF	0.01263	Nerve growth factor
97	IL2RA	0.01238	Interleukin 2 receptor subunit alpha
98	AQP5	0.01222	Aquaporin 5
99	CYP27B1	0.01222	Cytochrome P450 family 27 subfamily B member 1
100	NEFH	0.01218	Neurofilament heavy chain
101	CYP7A1	0.01202	Cytochrome P450 family 7 subfamily A member 1
102	MMP2	0.01186	Matrix metallopeptidase 2
103	APOE	0.01183	Apolipoprotein E
104	ICAM1	0.01177	Intercellular adhesion molecule 1
105	HLA-DRB3	0.01146	Major histocompatibility complex, class II, DR beta 3
106	CSF2	0.01134	Colony stimulating factor 2
107	IFNG	0.01133	Interferon gamma
108	ABCG2	0.01109	ATP binding cassette subfamily G member 2 (Junior blood group)
109	CXCL1	0.01109	C-X-C motif chemokine ligand 1
110	PPBP	0.01109	Pro-platelet basic protein
111	VCAM1	0.01056	Vascular cell adhesion molecule 1
112	APOA4	0.01035	Apolipoprotein A4
113	CXCL6	0.01035	C-X-C motif chemokine ligand 6
114	ESR1	0.01035	Estrogen receptor 1
115	MAP1LC3A	0.01035	Microtubule associated protein 1 light chain 3 alpha
116	IGBP1	0.01031	Immunoglobulin binding protein 1
117	APOB	0.00961	Apolipoprotein B
118	A1BG	0.00924	Alpha-1-B glycoprotein
119	TGFB1	0.00902	Transforming growth factor beta 1
120	SOD1	0.00887	Superoxide dismutase 1
121	TPO	0.00887	Thyroid peroxidase
122	PTPN22	0.00861	Protein tyrosine phosphatase non-receptor type 22
123	GJB6	0.00856	Gap junction protein beta 6
124	CALR	0.00848	Calreticulin
125	IL2	0.00847	Interleukin 2
126	AIF1	0.00832	Allograft inflammatory factor 1
127	CASP3	0.00832	Caspase 3
128	PRF1	0.00832	Perforin 1
129	CXCL12	0.00828	C-X-C motif chemokine ligand 12
130	CXCL14	0.00819	C-X-C motif chemokine ligand 14
131	AGT	0.00813	Angiotensinogen
132	CAT	0.00813	Catalase
133	C3	0.00796	Complement C3
134	CD79A	0.00795	CD79a molecule
135	BTD	0.00739	Biotinidase
136	DOCK8	0.00739	Dedicator of cytokinesis 8
137	DPP4	0.00739	Dipeptidyl peptidase 4
138	FGG	0.00739	Fibrinogen gamma chain
139	LAG3	0.00739	Lymphocyte activating 3
140	SERPINF1	0.00739	Serpin family F member 1
141	TNFRSF8	0.00739	TNF receptor superfamily member 8
142	SSB	0.00739	Small RNA binding exonuclease protection factor La
143	ITGAL	0.00665	Integrin subunit alpha L
144	BTG3	0.00615	BTG anti-proliferation factor 3
145	TNFSF13	0.00607	TNF superfamily member 13
146	CCL11	0.00591	C-C motif chemokine ligand 11
147	CCL17	0.00591	C-C motif chemokine ligand 17
148	CCR5	0.00591	C-C motif chemokine receptor 5
149	IL9	0.00591	Interleukin 9
150	CD163	0.00561	CD163 molecule
151	BCL2L1	0.00554	BCL2 like 1
152	F2	0.00524	Coagulation factor II, thrombin
153	AREG	0.00517	Amphiregulin
154	CCNA2	0.00517	Cyclin A2
155	SIRT1	0.00517	Sirtuin 1
156	LGI1	0.00499	Leucine rich glioma inactivated 1
157	PRL	0.00480	Prolactin
158	TNFRSF1A	0.00480	TNF receptor superfamily member 1A
159	HLA-C	0.00478	Major histocompatibility complex, class I, C
160	PTPRC	0.00462	Protein tyrosine phosphatase receptor type C
161	BRD2	0.00460	Bromodomain containing 2
162	NFASC	0.00451	Neurofascin
163	CD24	0.00443	CD24 molecule
164	CD6	0.00443	CD6 molecule
165	CHIA	0.00443	Chitinase acidic
166	CST3	0.00443	Cystatin C
167	GAPDH	0.00443	Glyceraldehyde-3-phosphate dehydrogenase
168	IRF8	0.00443	Interferon regulatory factor 8
169	PGAM1	0.00443	Phosphoglycerate mutase 1
170	PTS	0.00443	6-pyruvoyltetrahydropterin synthase
171	SCD5	0.00443	Stearoyl-coa desaturase 5
172	VDR	0.00443	Vitamin D receptor
173	CD27	0.00432	CD27 molecule
174	MAG	0.00425	Myelin associated glycoprotein
175	ATG5	0.00407	Autophagy-related 5
176	CCL2	0.00386	C-C motif chemokine ligand 2
177	ACE	0.00370	Angiotensin I converting enzyme
178	ACE2	0.00370	Angiotensin converting enzyme 2
179	ANAPC1	0.00370	Anaphase promoting complex subunit 1
180	ASAH1	0.00370	N-acylsphingosine amidohydrolase 1
181	B2M	0.00370	Beta-2-microglobulin
182	C1R	0.00370	Complement c1r
183	CD40	0.00370	CD40 molecule
184	CD80	0.00370	CD80 molecule
185	CLDN11	0.00370	Claudin 11
186	CTNNBL1	0.00370	Catenin beta like 1
187	CXCR4	0.00370	C-X-C motif chemokine receptor 4
188	CXCR5	0.00370	C-X-C motif chemokine receptor 5
189	DMD	0.00370	Dystrophin
190	ENG	0.00370	Endoglin
191	EOMES	0.00370	Eomesodermin
192	EXT2	0.00370	Exostosin glycosyltransferase 2
193	FOS	0.00370	Fos proto-oncogene, AP-1 transcription factor subunit
194	FOXK1	0.00370	Forkhead box K1
195	GPC5	0.00370	Glypican 5
196	HIF1A	0.00370	Hypoxia inducible factor 1 subunit alpha
197	HRAS	0.00370	Hras proto-oncogene, gtpase
198	IFNA2	0.00370	Interferon alpha 2
199	KRT83	0.00370	Keratin 83
200	MDK	0.00370	Midkine
201	MST1R	0.00370	Macrophage stimulating 1 receptor
202	MTFMT	0.00370	Mitochondrial methionyl-trna formyltransferase
203	NEFM	0.00370	Neurofilament medium chain
204	SIRT3	0.00370	Sirtuin 3
205	TFRC	0.00370	Transferrin receptor
206	TTR	0.00370	Transthyretin
207	ELANE	0.00368	Elastase, neutrophil expressed
208	IL5	0.00351	Interleukin 5
209	VTN	0.00351	Vitronectin
210	TF	0.00333	Transferrin
211	PTX3	0.00316	Pentraxin 3
212	NLRP3	0.00311	NLR family pyrin domain containing 3
213	CCL5	0.00296	C-C motif chemokine ligand 5
214	CD180	0.00296	CD180 molecule
215	CUBN	0.00296	Cubilin
216	CYP27A1	0.00296	Cytochrome P450 family 27 subfamily A member 1
217	FOXP3	0.00296	Forkhead box P3
218	ITGB1	0.00296	Integrin subunit beta 1
219	KLK4	0.00296	Kallikrein-related peptidase 4
220	KNG1	0.00296	Kininogen 1
221	SLC1A3	0.00296	Solute carrier family 1 member 3
222	RGMA	0.00294	Repulsive guidance molecule BMP co-receptor a
223	ABCB6	0.00259	ATP binding cassette subfamily B member 6 (Langereis blood group)
224	CD46	0.00259	CD46 molecule
225	CHIT1	0.00259	Chitinase 1
226	IL13	0.00259	Interleukin 13
227	CD68	0.00222	CD68 molecule
228	CD69	0.00222	CD69 molecule
229	CFP	0.00222	Complement factor properdin
230	CNTNAP2	0.00222	Contactin associated protein 2
231	CP	0.00222	Ceruloplasmin
232	F10	0.00222	Coagulation factor X
233	FCGRT	0.00222	Fc gamma receptor and transporter
234	LGALS3	0.00222	Galectin 3
235	MUSK	0.00222	Muscle associated receptor tyrosine kinase
236	NCAM1	0.00222	Neural cell adhesion molecule 1
237	ODC1	0.00222	Ornithine decarboxylase 1
238	PNMA2	0.00222	PNMA family member 2
239	RNASE3	0.00222	Ribonuclease A family member 3
240	SERPING1	0.00222	Serpin family G member 1
241	APP	0.00201	Amyloid beta precursor protein
242	AQP2	0.00185	Aquaporin 2
243	CCL24	0.00185	C-C motif chemokine ligand 24
244	HCRT	0.00185	Hypocretin neuropeptide precursor
245	HLA-B	0.00185	Major histocompatibility complex, class I, B
246	ITGAX	0.00185	Integrin subunit alpha X
247	B3GAT1	0.00148	Beta-1,3-glucuronyltransferase 1
248	CADM4	0.00148	Cell adhesion molecule 4
249	CCR3	0.00148	C-C motif chemokine receptor 3
250	CDKN2A	0.00148	Cyclin dependent kinase inhibitor 2A
251	CEBPZ	0.00148	CCAAT enhancer binding protein zeta
252	CLDN5	0.00148	Claudin 5
253	CNTNAP1	0.00148	Contactin-associated protein 1
254	CTSG	0.00148	Cathepsin G
255	DRD2	0.00148	Dopamine receptor D2
256	FCGR2A	0.00148	Fc gamma receptor iia
257	FGF2	0.00148	Fibroblast growth factor 2
258	GAD1	0.00148	Glutamate decarboxylase 1
259	HLA-A	0.00148	Major histocompatibility complex, class I, A
260	IL19	0.00148	Interleukin 19
261	IL21R	0.00148	Interleukin 21 receptor
262	MCAM	0.00148	Melanoma cell adhesion molecule
263	MPO	0.00148	Myeloperoxidase
264	MRS2	0.00148	Magnesium transporter MRS2
265	STAT4	0.00148	Signal transducer and activator of transcription 4
266	TNFSF4	0.00148	TNF superfamily member 4

To investigate the spatial association between imaging-derived maps (i.e. T2-hyperintense lesion probability map and the VBM-derived T-maps of gray and white matter atrophy) and candidate gene expression, we used the MENGA platform^13^ following published guidelines.^20^

To reduce variability and ensure reproducibility^20^ the transcriptomic data from the AHBA database underwent the following steps:

1. Representative probe selection to index expression for a gene: one probe was collected as representative of a specific gene transcript for all donors; 71% of genes in the AHBA were measured with at least two probes. Thus, probe selection was performed by evaluating the distribution of the expression values and the one with the most symmetric and least skewed profile was selected to avoid the nonlinearity effect on the microarray measures.^13^
2. Normalization of the expression measure by inter-individual differences (https://github.com/BMHLab/AHBAprocessing): to address donor-specific effects and remove the inter-individual differences in expression measure, each gene expression value was normalized across brain regions separately in each donor to reflect its relative expression across different brain regions. Then, Z-score normalization was obtained as follows

$$z_{score}=\frac{x_{i}-\bar{x}}{\sigma }$$

where $\bar{x}$ and σ are the mean and standard deviation, respectively, and $x_{i}$ is the expression value of a specific gene in a single sample.

Image maps in the MNI space were then resampled into the AHBA coordinates, separately for each donor, to obtain image-to-sample spatial correspondence according to MNI coordinates of each sample provided by the AHBA. Image data were normalized to z-scores and each image sample was estimated as the average of the voxels within a 3D window of a specified size (here set to 5 mm) centered on the MNI coordinates of the genomic sample.

Spatial association between imaging and genomic data were then assessed with MENGA platform using a weighted multiple regression, with the directionality of the imaging-genomic data correlation provided as well.

The chance likelihood, a measure of cross-correlation reliability, was estimated as^13^

$$chance\;likelihood=\frac{number\;of\;instances\;(R^{2}>R_{or}^{2})}{number\;of\;bootstraps}$$

where $R_{or}^{2}$ is the value of the coefficient obtained from the real data, and $R^{2}$ is that obtained using the bootstrapped genomic data. It returns the probability that genomic data are unrelated to the image values.

Thus, smaller values of the chance likelihood denote a higher reliability of the obtained results. In this work, we set a cut-off < 0.05 likelihood to assess significant spatial associations between brain damage and gene expression.

*Enrichment analysis*. To better understand functionalities, we performed a functional-enrichment analysis according to the Reactome pathway database^21^ using the g:Profiler platform (https://biit.cs.ut.ee/gprofiler/gost).^22^ Briefly, the list of the genes significantly associated with the different types of structural damage was used as input to the platform, which identifies overrepresented biological processes through an over-representation analysis (threshold: 5–2500 genes per enriched pathway). Only significant results at Bonferroni-corrected threshold (*p* < 0.05) were retained. The Cytoscape software version 3.9.0 was used to visualize the interactions between the enriched pathways.^23^

### Statistical analysis

Demographic, clinical, and conventional MRI variables were compared with Mann–Whitney *U*-test or independent samples *t*-test (quantitative variables, based on normality assumption) or Pearson’s Chi-square test (categorical variables).

## Results

### Demographic, clinical, and conventional MRI features of the study population

AQP4 + NMOSD patients and HC had similar age, but female sex was more common in patients, as per disease epidemiology. The proportion of participants acquired on each scanner was balanced in between patients and HC. AQP4 + NMOSD patients had whole brain and gray matter atrophy (Table 3).

**Table 3.** Demographic, clinical, and conventional MRI features of the study population. Quantitative variables are presented as mean (standard deviation) or median (interquartile range), according to the normality assumption; categorical variables are presented as frequency (percentage). If not otherwise specified, *p* values refer to Mann–Whitney *U*-test (quantitative variables) or Pearson’s Chi-square test (categorical variables).

	AQP4 + NMOSD (*n* = 80)	HC (*n* = 94)	*p* value
Demographic features			
Age (SD; years)	45.2 (13.8)	45.3 (13.3)	0.95^a^
Female sex	68 (85%)	66 (70%)	**0.02**
Scanners			
Scanner #1 (Milan, Intera)	31 (39%)	36 (38%)	0.53
Scanner #2 (Milan, Ingenia)	9 (11%)	16 (17%)	
Scanner #3 (Belgrade)	40 (50%)	42 (45%)	
Disease duration (IQR; years)	3.5 (0–34)	–	–
History of optic neuritis	53 (66%)	–	–
Number of optic neuritis^b^	2 (1–4)	–	–
History of myelitis	69 (86%)	–	–
Number of myelitis	2 (1–3)	–	–
History of diencephalic involvement	6 (8%)	–	–
History of brainstem/area postrema syndrome	14 (18%)	–	–
EDSS (IQR)	4.0 (1.0–9.0)	–	–
Conventional MRI measures			
T2-hyperintense lesion volume (IQR; mL)	0.25 (0.06–1.25)	0.00 (0.00–0.36)	**0.02**
Normalized brain volume (SD; mL)	1468 (189)	1537 (96)	**<0.001**
Normalized gray matter volume (SD; mL)	729 (108)	776 (74)	**<0.001**
Normalized white matter volume (SD; mL)	737 (116)	759 (85)	0.34

AQP4 + NMOSD: aquaporin-4-IgG-positive neuromyelitis optica spectrum disorder; EDSS: expanded disability status scale; HC: healthy controls; SD: standard deviation; IQR: interquartile range.

Bold is for significant values.

a Independent sample *t*-test.

b Only patients with a history of optic neuritis.

### T2-hyperintense lesion probability map

**Figure 1.**
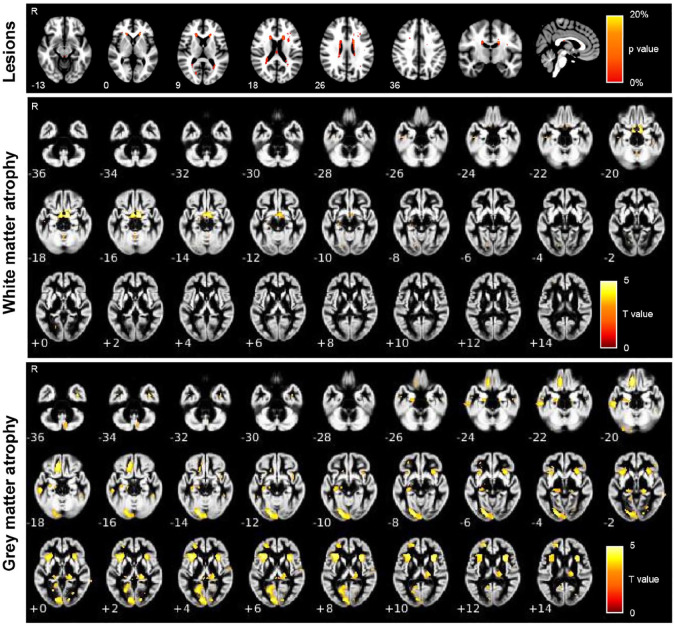
Spatial location of T2-hyperintense lesions, gray matter atrophy, and white matter atrophy in AQP4 + NMOSD patients. Atrophy T maps refer to age-, sex-, scanner-, and V-scaling-adjusted full factorial models (AQP4 + NMOSD vs HC, *p* < 0.001). Numbers refer to the z coordinates according to the MNI system. Abbreviations: R: right; MNI: Montreal Neurological Institute.

Brain T2-hyperintense lesions were detected in 59/80 patients (74%) and were mainly located in the periventricular white matter. Additional locations were the subcortical white matter, corpus callosum, and the periaqueductal gray (Figure 1).

### Gray and white matter atrophy—VBM

Compared with HC, AQP4 + NMOSD patients had significant white matter atrophy selectively involving the optic tracts, bilaterally (Figure 1). Gray matter atrophy was significant in the visual cortex (lingual gyrus, calcarine fissure), insula, prefrontal cortex (orbitofrontal gyrus), and lateral geniculate nuclei of the thalami (Figure 1). No voxels of significant atrophy were found in HC compared with AQP4 + NMOSD patients.

### Spatial association between gene expression and brain damage in AQP4 + NMOSD patients

**Figure 2.**
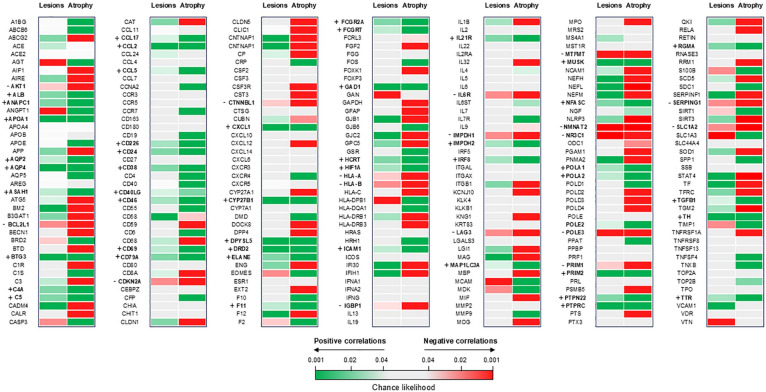
Heatmap of spatial association between gene expression, T2-hyperintense lesions, and gray matter atrophy in AQP4 + NMOSD patients. Positive correlations (i.e. the highest the expression of the gene, the highest the probability of a focal lesion/T value of gray matter atrophy) are color-coded green, while negative correlations (i.e. the highest the expression of the gene, the lowest the probability of a focal lesion/T value of gray matter atrophy) are color-coded red. Nonsignificant correlations are light gray. Genes positively correlated with both lesions and gray matter atrophy are shown in bold and indicated by a+; genes negative correlated with both lesions and gray matter atrophy are shown in bold and indicated by a-.

Since white matter atrophy selectively involved the optic tracts, which are not sampled in the AHBA, the VBM-derived map of white matter atrophy was not included in this analysis, which therefore, was run on the T2-hyperintense lesion probability maps and the VBM-derived map of gray matter atrophy only. Significant associations are reported in Figure 2.

Both T2-hyperintense lesions and gray matter atrophy were positively associated with AQP4 expression. When analyzing the targets of the Food and Drug Administration (FDA)-approved treatments for AQP4 + NMOSD, we found that a higher expression of complement factors (i.e. C5 and C4a) was also associated with both T2-hyperintense lesions and gray matter atrophy. CD19 expression was positively associated with gray matter atrophy, while components of the IL6 pathway showed mixed or nonsignificant associations.

A few genes resulted negatively associated with both T2-hyperintense lesions and atrophy, including some involved in immune system/complement inhibition (e.g. LAG3, SERPING1) and axonal survival (e.g. NMNAT2).

### Gene enrichment analysis

**Figure 3.**
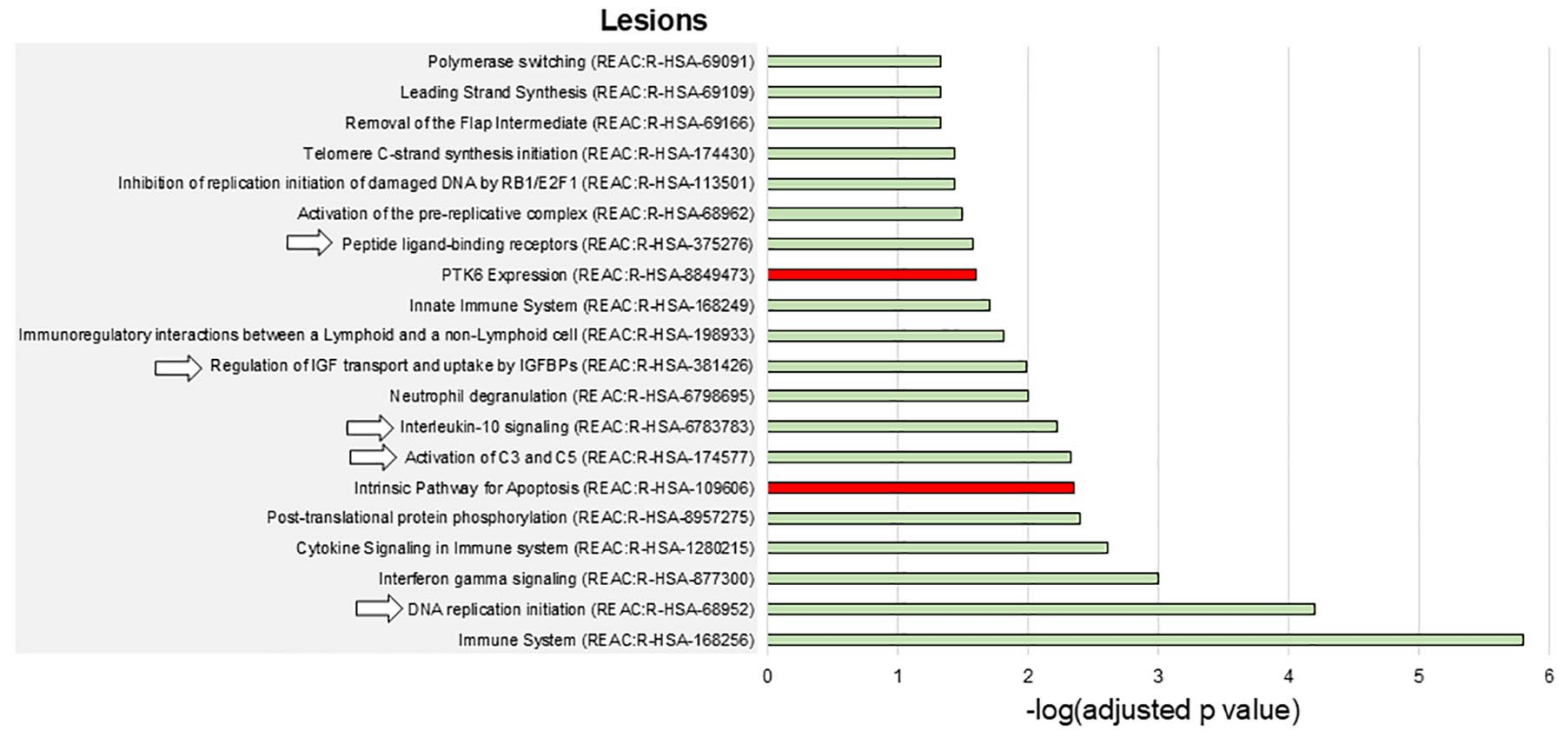
Enriched biological pathways associated with T2-hyperintense lesions in AQP4 + NMOSD. The barcharts show the enriched biological pathways positively (green bars) or negatively (red bars) associated with T2-hyperintense lesions in AQP4 + NMOSD. Numbers on the *x* axes are -log(Bonferroni-adjusted *p* values) of the enrichment analysis. The pathways indicated by arrows are also positively associated with the presence of gray matter atrophy. None of the pathways negatively associated with T2-hyperintense lesions was also negatively associated with gray matter atrophy. Abbreviations: REAC: reactome pathways database.

**Figure 4.**
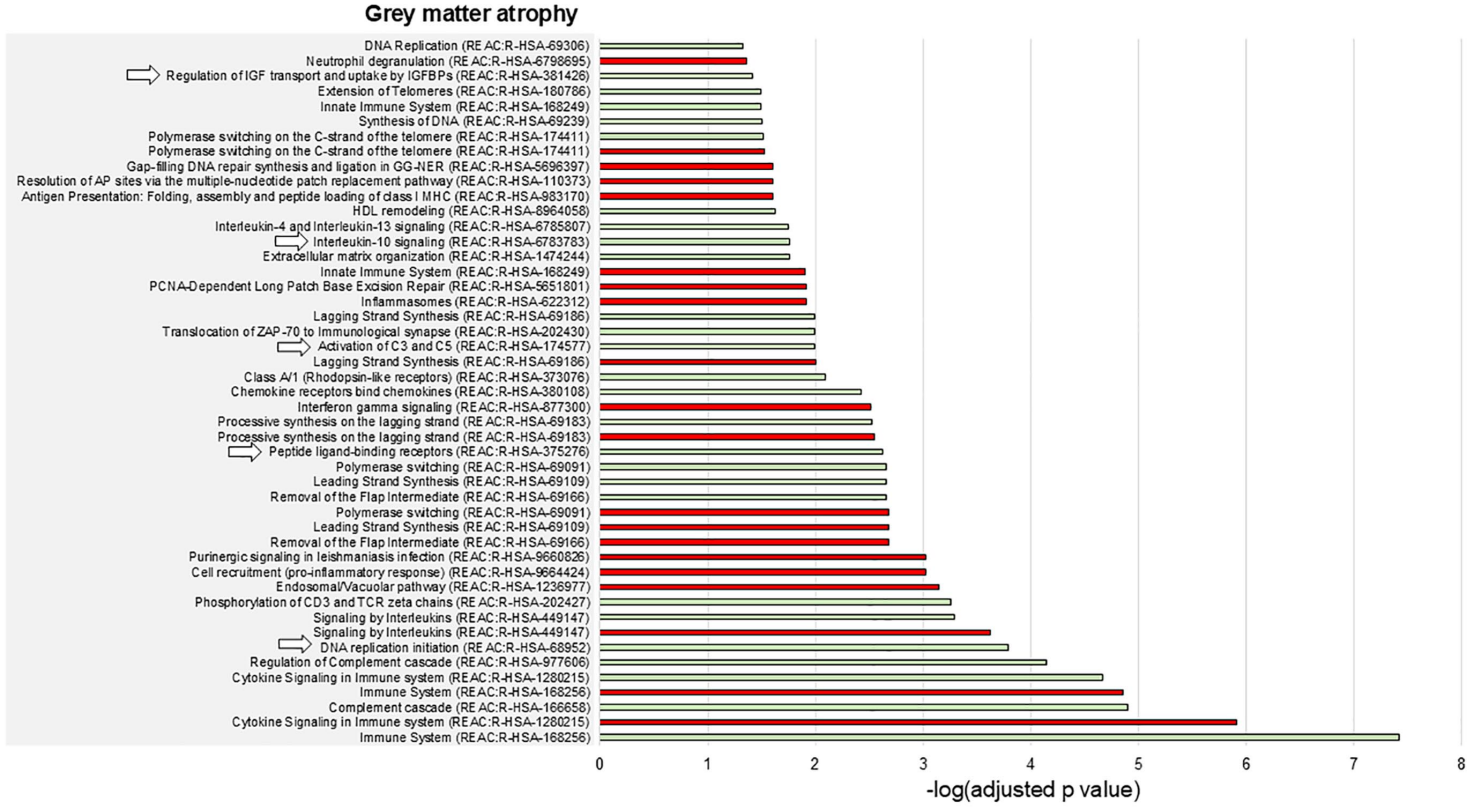
Enriched biological pathways associated with gray matter atrophy in AQP4 + NMOSD. The barcharts show the enriched biological pathways positively (green bars) or negatively (red bars) associated with gray matter atrophy in AQP4 + NMOSD. Numbers on the *x* axes are -log(Bonferroni-adjusted *p* values) of the enrichment analysis. The pathways indicated by arrows are also positively associated with the presence of T2-hyperintense lesions. None of the pathways negatively associated with gray matter atrophy was also negatively associated with T2-hyperintense lesions. Abbreviations: REAC: reactome pathways database.

Overall, the enrichment analysis disclosed a limited number of enriched biological processes spatially associated with the presence of T2-hyperintense lesions (Figure 3), while gray matter atrophy was associated with a high number of biological processes, mainly related to DNA synthesis and repair (Figure 4).

Complement activation, the regulation of IGF and uptake by IGFBPs, and IL10 signaling were positively associated with both T2-hyperintense lesions and gray matter atrophy (Figures 3 and 4). Broader processes (i.e. DNA replication initiation and the response to peptide-ligand binding receptors) showed a positive association with both types of damage as well.

**Figure 5.**
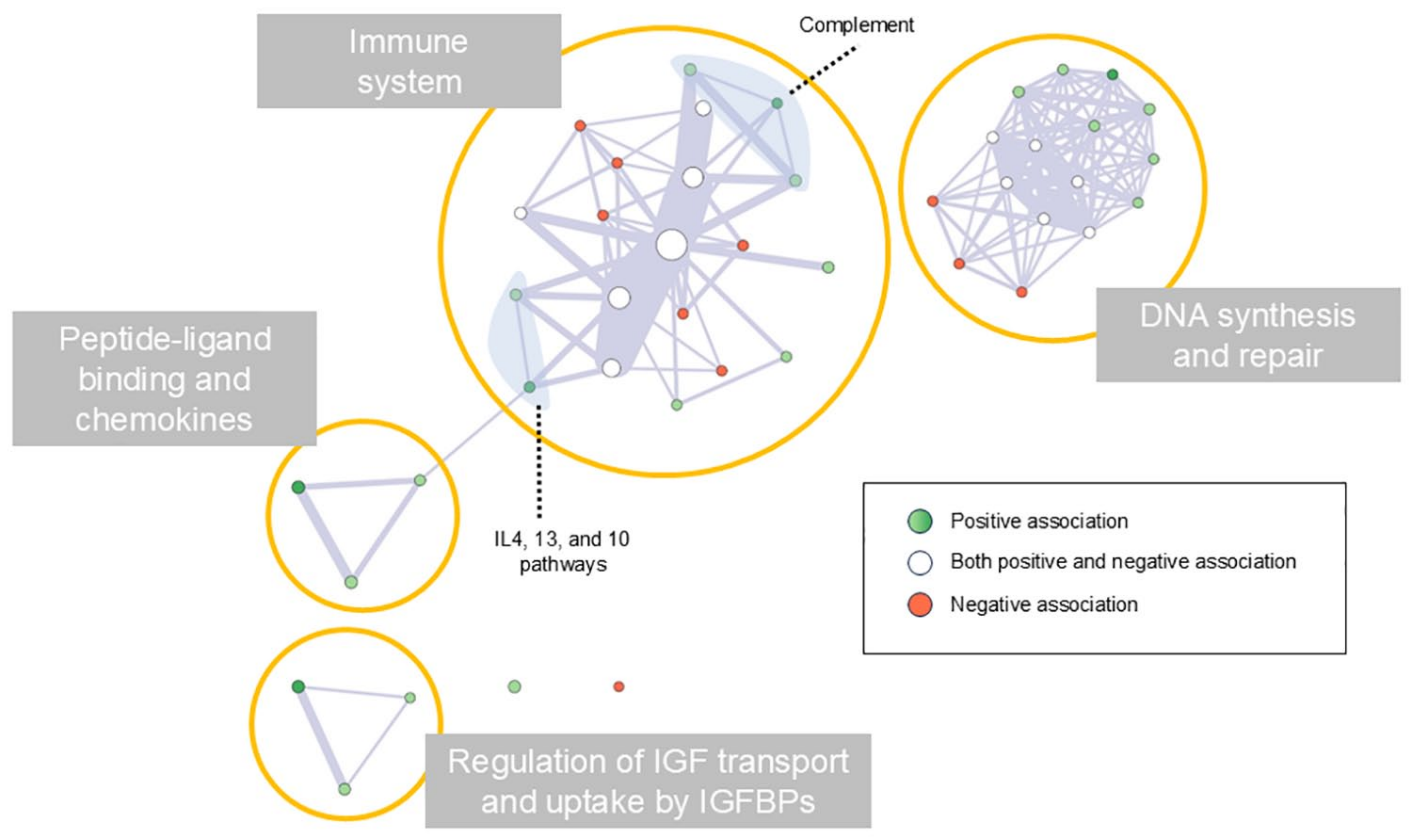
Network of the enriched biological pathways involved in brain damage of AQP4 + NMOSD. Each node represents one of the enriched biological pathways, and their size represents the size of the pathway. Edge width represents the number of genes shared by connected pathways. Green nodes indicate enriched pathways positively associated with the development of brain damage (dark green indicates the pathways involved in both T2-hyperintense lesions and gray matter atrophy formation). Red nodes indicate enriched pathways negatively associated with the development of brain damage. White nodes indicate enriched pathways showing both positive and negative associations with brain damage. Abbreviations: IGF: insulin-like growth factor; IGFBPs: insulin-like growth factor-binding proteins; IL: interleukin; REAC: reactome pathways database.

The network of the enriched biological processes disclosed four main clusters: pathways within (1) the immune system, (2) mechanisms of DNA synthesis and repair, (3) processes of peptide-ligand binding and chemokines, and (4) regulation of the insulin-like growth factor (IGF) transport and uptake by insulin-like growth factor binding proteins (IGFBPs; Figure 5).

## Discussion

The understanding of disease pathogenesis is a pivotal step for the identification of tailored treatment strategies. In this proof-of-concept study, we used a joint methodology merging quantitative measures of brain damage and the a-priori mapping of gene expression in the human brain to assess in-vivo correlates of AQP4 + NMOSD, an autoimmune neurological disorder of known pathophysiology.

Our results confirmed the key role of AQP4 expression for the presence of brain lesions and atrophy, but were also able to identify the relevant biological pathways, including the activation of the complement cascade, the regulation of IGF and uptake by IGFBPs, and IL-4, -13, and -10 signaling.

The activation of the complement cascade and the recruitment of granulocytes at the site of damage were already identified as key steps of AQP4 + NMOSD pathophysiology, as demonstrated in pathology,^2^ explaining the spatial association with eosinophils chemoattractants such as IL-4 and IL-13. Neutrophil degranulation was associated with lesion formation as well.

Treatments inhibiting the complement cascade, such as eculizumab and ravulizumab, are highly effective in AQP4 + NMOSD,^7,24^ and our results confirm the central role of complement in the development of brain damage. As expected, results were less explanatory when considering targets of treatments acting outside the CNS, such as IL-6 and CD19. This approach merges local brain transcriptomic and the topography of brain damage. Therefore, it is not suitable for analyzing dynamic phenomena or effector pathways occurring outside the CNS. Along this line, it is important to mention that neuroinflammation and tissue damage as well as tissue healing are able to trigger changes in tissue gene expression.^25,26^ Such changes cannot be underpinned by our methodology due to its cross-sectional nature and to the use of a static atlas of gene expression derived from healthy donors, where these changes are not expected.

In addition, the MRI scans of our patients were acquired during disease remission. Recent studies demonstrated that about 5% of brain lesions completely resolve and almost invariably reduce in size after acute AQP4 + NMOSD attacks.^27^ Therefore, we might have missed associations with acute inflammatory lesions.

We also found a significant spatial association between IL-10 signaling and brain damage, but the literature on this topic is controversial. Despite the classical interpretation of IL-10 as an anti-inflammatory molecule, the recent SARS-CoV-2 pandemic has questioned this hypothesis showing that high IL-10 levels were associated with a more severe disease course.^28^

Brain damage was also associated with the regulation of IGF transport and uptake by IGPBPs. This process was not previously investigated in AQP4 + NMOSD, but experimental data highlighted that IGF dysregulation (i.e. reduction) fuels autoimmunity through T reg unbalance,^29^ and hampers remyelination.^30^ Astrocytes produce IGF in response to brain injury.^31^ Therefore, this protective mechanism might be deficient in AQP4 + NMOSD.

In general, the biological processes colocalizing with brain lesions (i.e. a proxy of focal inflammatory damage) were specific of AQP4 + NMOSD pathogenesis. In contrast, gray matter atrophy (i.e. a proxy of neurodegenerative damage) was associated with many pathways, often nonspecific and sometimes with unexpected directions. In our cohort, (where most patients had prior history of optic neuritis) brain atrophy mainly involved structures within the visual pathways such as the optic tracts and the occipital cortex, supporting the secondary nature of this phenomenon. Such distribution overlaps in the large majority of studies (including multicentric studies) exploring this aspect in AQP4 + NMOSD, supporting its generalizability.^11^ Therefore, although the presence of AQP4 and the consequent trigger of the complement cascade might determine the development of local atrophy as well, similarly to what was observed in the spinal cord,^32^ our interpretation is that this type of imaging analysis is not suitable for the study of secondary mechanisms of damage, because their localization is better explained by the anatomy of functional pathways (such as the visual pathway) rather than by local tissue gene expression.

Finally, a few potentially protective genes, emerged. Among them, those exerting a negative effect on immune pathways activation, such as the T-cell inhibitor lymphocyte activation gene 3 (LAG3) and the complement inhibitor serpin family G member 1 (SERPING1) seem particularly promising. LAG3 is a key warrantor of immuno-tolerance, to the point that inhibitory monoclonal antibodies are currently approved for cancer immunotherapy.^33^ The gene SERPING1 encodes the C1 esterase inhibitor, which inhibits both the classical and lectin complement pathways. Its mutations are associated with hereditary angioedema, highlighting its central role in complement pathways regulation.^34^ Another interesting gene is the nicotinamide mononucleotide adenylyltransferase 2 (NMNAT2), which localizes in axons and inhibits wallerian degeneration. A recent study has proved it as a potential therapeutic target against retinal ganglion cell neurodegeneration.^35^ However, future research is needed to support their potential therapeutic role in AQP4 + NMOSD.

Moving to the main limitations of the study, we acknowledge the cross-sectional design and the abovementioned nuisance effect of lesion resolution. Unfortunately, biological samples of patients were unavailable at our facility, hence a biological confirmation of this approach is lacking. However, our approach was able to identify complement activation as one of the most relevant biological processes associated with brain damage in AQP4 + NMOSD, which was never shown before.

Overall, we believe that the importance of this study is not to be found in the results per se, but in its translational potential. AQP4 + NMOSD represents a unique research scenario where the pathogenetic element and pathophysiology are already known and proven by the efficacy of tailored treatments. Therefore, it can be used as a research model, where confirmatory results support its application to other autoimmune disorders.

Future investigations will clarify the translational power of this approach, possibly integrating results with patients’ genotyping, the measurement of serum or cerebrospinal fluid (CSF) cytokines, and biomarkers of neuronal loss.

## Supplemental Material

sj-docx-1-msj-10.1177_13524585241307154 – Supplemental material for Use of brain MRI and gene expression atlases to reconstruct the pathophysiology of autoimmune neurological disorders: The proof-of-concept of NMOSD Supplemental material, sj-docx-1-msj-10.1177_13524585241307154 for Use of brain MRI and gene expression atlases to reconstruct the pathophysiology of autoimmune neurological disorders: The proof-of-concept of NMOSD by Laura Cacciaguerra, Loredana Storelli, Elisabetta Pagani, Paolo Preziosa, Sharlota Mesaros, Vittorio Martinelli, Lucia Moiola, Marta Radaelli, Jovana Ivanovic, Olivera Tamas, Jelena Drulovic, Massimo Filippi and Maria A Rocca in Multiple Sclerosis Journal
