# Supplementary material for: Use of brain MRI and gene expression atlases to reconstruct the pathophysiology of autoimmune neurological disorders: The proof-of-concept of NMOSD
Source: Mult Scler. 2024 Dec 31;31(2):140–58. doi: 10.1177/13524585241307154 (PMC11789429; doi:10.1177/13524585241307154)
Supplement: sj-docx-1-msj-10.1177_13524585241307154 – Supplemental material for Use of brain MRI and gene expression atlases to reconstruct the pathophysiology of autoimmune neurological disorders: The proof-of-concept of NMOSD [file sj-docx-1-msj-10.1177_13524585241307154.docx]

MS Journal Appendix for MRI methodology

| **Hardware** | |
| --- | --- |
| Field strength | 3.0 and 1.5 T |
| Manufacturer | Philips |
| Model | Philips Intera; Philips Ingenia; Philips Achieva |
| Coil type  (e.g. head, surface) | Head |
| Number of coil channels |  |

| **Acquisition sequence** | | |
| --- | --- | --- |
| Type  (e.g. FLAIR, DIR, DTI, fMRI) | T2: FLAIR, dual echo TSE; T1: FFE, MPRAGE, TFE | |
| Acquisition time |  | |
| Orientation |  | |
| Alignment  (e.g. anterior commissure/poster commissure line) | Axial; Sagittal | |
| Voxel size |  | |
| TR | See Table 1 | |
| TE | See Table 1 | |
| TI | See Table 1 | |
| Flip angle |  | |
| NEX |  | |
| Field of view |  | |
| Matrix size | See Table 1 | |
| Parallel imaging | Yes | No |
| If used, parallel imaging method:  (e.g. SENSE, GRAPPA) |  | |
| Cardiac gating | Yes | No |
| If used, cardiac gating method:  (e.g. PPU or ECG) |  | |
| Contrast enhancement | Yes | No |

| **Acquisition sequence** | |
| --- | --- |
| If used, provide name of contrast agent, dose and timing of scan post-contrast administration |  |
| Other parameters: |  |

| **Image analysis methods and outputs** | |
| --- | --- |
| ***Lesions*** | |
| Type  (e.g. Gd-enhancing, T2-hyperintense, T1-hypointense) | T2-hyperintense |
| Analysis method | Local thresholding segmentation technique |
| Analysis software | Jim 7.0, Xinapse Systems Ltd., Colchester, UK |
| Output measure  (e.g. count or volume [ml]) | T2-lesion volume |
| ***Tissue volumes*** | |
| Type  (e.g. whole brain, grey matter, white matter, spinal cord) | Whole brain |
| Analysis method | SIENAx |
| Analysis software | FSL |
| Output measure  (e.g. absolute tissue volume in ml, tissue volume as a fraction of intracranial volume, percentage change in tissue volumes) | Normalized brain volume |
| ***Tissue measures (e.g. MTR, DTI, T1-RT, T2-RT, T2*, T2’, ^1^H-MRS, perfusion, Na)*** | |
| Type  (e.g. whole brain, grey matter, white matter, spinal cord, normal-appearing grey matter or white matter) |  |
| Analysis method |  |
| Analysis software |  |
| Output measure |  |
| ***Other MRI measures (e.g. functional MRI)*** | |
| Type  (e.g. whole brain, grey matter, white matter, spinal cord, normal-appearing grey matter or white matter) | Grey matter |
| Analysis method | fMRI |
| Analysis software | SPM12 |
| Output measure | resting-state functional connectivity |

**Other analysis details:**

seed-based functional connectivity
